# A LEA model peptide protects the function of a red fluorescent protein in the dry state

**DOI:** 10.1016/j.bbrep.2018.11.006

**Published:** 2018-11-26

**Authors:** Takao Furuki, Tatsuya Niwa, Hideki Taguchi, Rie Hatanaka, Takahiro Kikawada, Minoru Sakurai

**Affiliations:** a Center for Biological Resources and Informatics, Tokyo Institute of Technology, B-62 4259, Nagatsuta-cho, Midori-ku, Yokohama 226-8501, Japan; b Cell Biology Center, Institute of Innovative Research, Tokyo Institute of Technology S2-19, 4259 Nagatsuta, Midori-ku, Yokohama 226-8503, Japan; c Molecular Biomimetics Research Unit, Division of Biotechnology, Institute of Agrobiological Sciences, National Institute of Agriculture and Food Research Organization, Ohwashi 1-2, Tsukuba 305-8634, Japan

**Keywords:** G3LEA, group 3 late embryogenesis abundant, RFP, red fluorescent protein, LEA protein, Anhydrobiosis, Trehalose, Desiccation tolerance, Dry preservation

## Abstract

We tested whether a short model peptide derived from a group 3 late embryogenesis abundant (G3LEA) protein is able to maintain the fluorescence activity of a red fluorescent protein, mKate2, in the dry state. The fluorescence intensity of mKate2 alone decreased gradually through repeated dehydration-rehydration treatments. However, in the presence of the LEA model peptide, the peak intensity was maintained almost perfectly during such stress treatments, which implies that the three dimensional structure of the active site of mKate2 was protected even under severe desiccation conditions. For comparison, similar experiments were performed with other additives such as a native G3LEA protein, trehalose and BSA, all of whose protective abilities were lower than that of the LEA model peptide.

## Highlights

• We prepared a 22-mer model peptide of a group-3 LEA protein.
• The fluorescent peak of a red fluorescent protein was almost lost on drying.
• The model peptide suppressed such desiccation-induced damage.
• This indicates that the 3D structure of the fluorophore was protected.
• The peptide exhibited the highest protective effect among the reagents tested.

## 1 Introduction

Late embryogenesis abundant (LEA) proteins are well-characterized hydrophilic proteins that upregulate in response to environmental stresses such as desiccation, freezing, and high salinity [1], [2], [3], [4]. They have been classified into several groups based upon their gene expression pattern and amino acid sequence [1], [2], [3], [4] and most examples discovered so far in animals such as the sleeping chironomid (*Polypedilum vanderplanki*) are group 3 LEA (G3LEA) proteins [4], [5]. These proteins have several tandem repeats of loosely conserved 11-mer motifs [3], [4] that have charged residues such as Lys, Glu, or Asp in positions 3, 6, 7, 8 and 11 [6]. G3LEA proteins are also intrinsically disordered proteins (IDPs) [1], [2], [3], [4]: they are disordered in the hydrated state, but become more ordered upon dehydration, when they predominantly form α-helical structures [7], [8], [9], [10], [11], [12].

G3LEA proteins have been reported to protect biological molecules from the effects of desiccation stress; for example, they prevent membrane fusion [13], [14], [15] and protein aggregation [16], [17], [18], [19], and preserve enzyme activity [20], [21], [22], [23], [24], [25]. It is of great interest to elucidate how the repeated 11-mer motifs mentioned above contribute to these biological functions. For this purpose, we have studied the structural and functional properties of chemically synthesized 22-mer and 44-mer peptides, named PvLEA-22 and PvLEA-44, respectively, which comprise two and four tandem repeats of the 11-mer motifs in G3LEA proteins originating from the sleeping chironomid [26], [27], [28], [29], [30], [31]. When dried, both LEA model peptides were found to adopt the glassy state and remain in this state at temperatures up to 100 °C; they are also capable of reinforcing the glassy matrix of the non-reducing disaccharide, trehalose [26], [27]. Like native G3LEA proteins, both peptides serve as good protective reagents for proteins and liposomes in the dry state [28], [29], [30], [31].

Several mechanisms have been proposed for the function of G3LEA proteins: cytoskeleton formation [1], molecular shielding [32], ion sequestration [6] and vitrification [1], [7]. Of these, the molecular shielding mechanism may best explain the anti-aggregation effect of G3LEA proteins: they act as a physical barrier between target biological molecules and thereby decrease the collision frequency of potentially aggregating species in cells [32]. However, it is unclear whether the molecular shielding effect alone is effective at preventing desiccation-induced intramolecular damage in a target protein. The fact that G3LEA proteins and their model peptides [20], [21], [22], [23], [24], [25], [30] are able to maintain the catalytic activity of dried enzymes implies that they protect the tertiary structures of the targets, at least of their active sites, during drying. To address this issue more deeply, it would be desirable to use a target protein whose secondary structures are stable in the dry state and for which tertiary structural changes can be detected in a simple and sensitive way. Fluorescent proteins are one of the best candidates for this purpose. Chakrabortee et al. used a red fluorescent protein (RFP), mCherry, as a target and found that a native G3LEA protein, AavLEA1 from an anhydrobiotic nematode (*Aphelenchus avenae*), had only a limited protective effect on this target in the dry state [32]. Thus, there is still no clear answer to the above question.

In the present study, we selected another RFP, mKate2 [33], as a target and performed a comparative test of the desiccation protective activity of several protectants, including PvLEA-22, a native G3LEA protein, bovine serum albumin (BSA) and trehalose. The results of fluorescence emission measurements indicate that PvLEA-22 almost completely inhibits intramolecular damage of mKate2 in the dry state and is superior to the other protectants.

## 2 Materials and methods

### 2.1 Protectant preparation

PvLEA-22 consists of two tandem repeats of the 11-mer motif, AKDGTKEKAGE. For comparison, we prepared another 22-mer peptide, referred to as the scrambled peptide, whose composition is identical to that of PvLEA-22 but whose sequence was scrambled: AKEKEGTDKAGGAKDTGEKEKA. These peptides were synthesized by Funakoshi Co. (Tokyo, Japan). A G3LEA protein originating in the African sleeping chironomid, PvLEA4, was obtained in recombinant form after expression in *Escherichia coli*. Details of PvLEA4 production and purification are described in Ref. [12]. BSA was purchased from Sigma-Aldrich Co (St. Louis, MO). Trehalose was kindly gifted by Hayashibara Co. (Okayama, Japan).

### 2.2 Recombinant mKate2 preparation

**Fig. 1:**
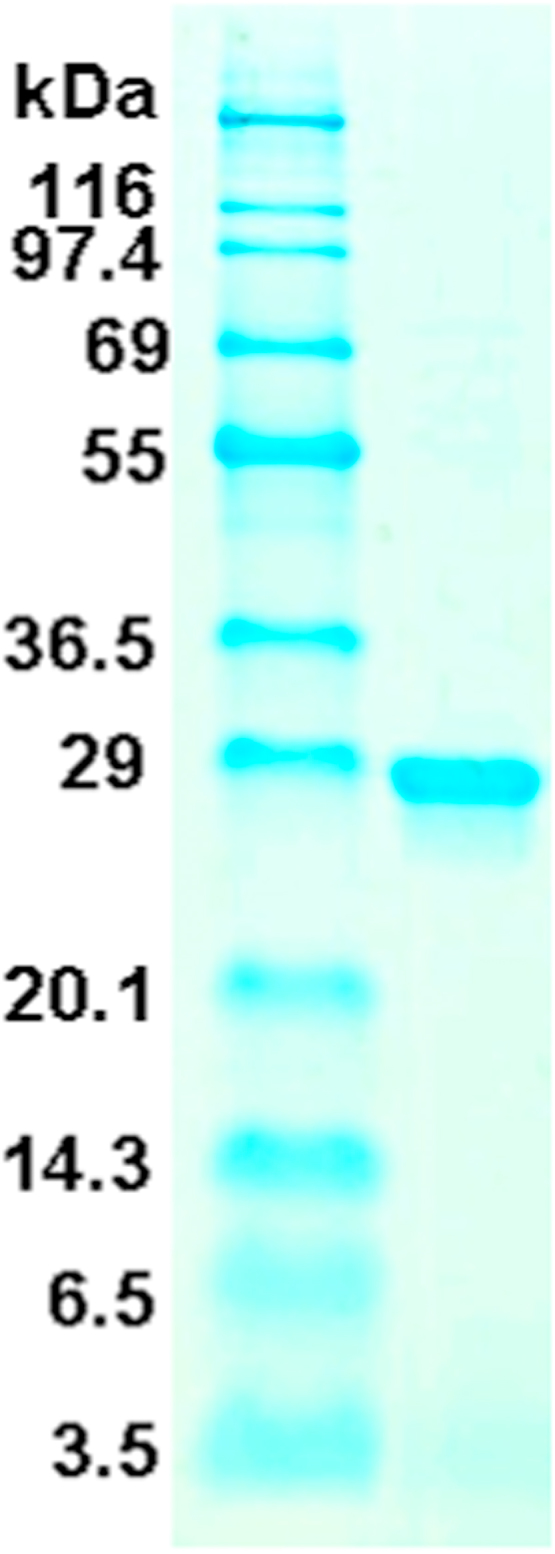
SDS-PAGE analysis of purified recombinant mKate2. Molecular masses of standard proteins are indicated on the left: insulin B chain (bottom), aprotinin, lysozyme, trypsin, carbonic anhydrase, lactic dehydrogenase, glutamic dehydrogenase, bovine serum albumin, phosphorylase b, and β-galactosidase (top).

mKate2 (Evrogen, Moscow, Russia) was recombinantly obtained in the following way. Its C-terminal 6xHis-tagged protein was overexpressed in *E. coli* BL21 (DE3). The cells were resuspended in buffer A [25 mM Hepes-KOH (pH 7.4), 1 M NH_4_Cl, 5 mM MgCl_2_, and 7 mM 2-mercaptoethanol] and disrupted by sonication. The cell lysate was centrifuged at 50,000 × *g* for 45 min in a JA-30.50 Ti rotor (Beckman Coulter, Brea, CA). The supernatant was collected and applied to a HisTrap column using an AKTA purifier system (GE Healthcare, Chicago, IL) and the 6×His-tagged mKate2 was eluted using the buffer A with a linear gradient of 5–60% buffer B [25 mM Hepes-KOH (pH 7.4), 100 mM KCl, 5 mM MgCl_2_, 500 mM imidazole, and 7 mM 2-mercaptoethanol]. The protein solution obtained was then passed through a NAP-5 column (Sephadex G-25, GE Healthcare) filled with Milli-Q water and the fraction colored red was collected. The purity of mKate2 in the fraction was confirmed by SDS-PAGE and Coomassie blue straining (Fig. 1). The mKate2 concentration was measured at its characteristic absorption wavelength of 588 nm (absorption coefficient = 62,500 M^−1^ cm^−1^) [33].

### 2.3 Desiccation tolerance assay

An mKate2 solution with a concentration of 1.15 × 10^−4^ M (3 mg/mL) was prepared in 20 mM Tris-HCl buffer (pH 7.5). The Tris-buffer was selected in accordance with a previous study [33]. The protective activity of each protectant was tested by adding it individually to this mKate2 solution before drying. The concentrations of PvLEA-22, the scrambled peptide and trehalose relative to mKate2 were determined by taking into account their molecular surface area (MSA). According to the X-ray structure of mKate2 (PDBID 3BXB), this molecule forms a cylindrical shape with a diameter and length of 3 nm and 4 nm, respectively. Based on this, the MSA of mKate2 is estimated to be 52 nm^2^. The MSAs of PvLEA-22 and trehalose are 4.3 nm^2^ [29], [30] and 0.69 nm^2^ [34], respectively. Therefore, the minimal molar ratio of the LEA model peptide needed to cover the entire surface of the mKate2 molecule is about 12. A native LEA protein, PvLEA4, includes seven true copies of the 11-mer motif [12]. To compare its protective effect with that of PvLEA-22 on the same 11-mer motif concentration basis, the molar ratio of PvLEA4 relative to mKate2 was determined to be 3.4. The molar ratio of BSA relative to mKate2 was set to be the same as PvLEA4, i.e. 3.4. For trehalose, two different concentrations were tested. One was the minimum amount to cover the entire surface of mKate2, for which the sugar/ mKate2 molar ratio was 74. The other represented a 10-fold excess over the minimum amount, i.e. a molar ratio of 740.

Twenty μL of each mKate2/protectant mixed solution prepared above was placed in an Eppendorf tube and dried in vacuum desiccator at room temperature for one day. The resulting dried sample was rehydrated with 20 μL Milli-Q water. Hereafter, this drying-rehydration treatment is defined as one cycle. We performed spectroscopic measurements after one, three or five cycles of such a treatment for each mKate2/protectant sample. Absorption spectra were measured with a spectrophotometer (U-2900; Hitachi Instruments, Hitachi, Japan). Fluorescence emission spectra were recorded with a fluorometer (FP-6500; JASCO, Tokyo, Japan) at an excitation wavelength of 588 nm and emission wavelength of 620 nm. Circular dichroism (CD) spectra were measured with a spectropolarimeter (J-1100; JASCO, Tokyo, Japan) over a 190–250 nm range at room temperature.

The results of the fluorescent intensity measurements were subjected to statistical analysis by 2-way ANOVA using Prism version 6 (GraphPad Software, La Jolla, CA).

## 3 Results and discussion

**Fig. 2:**
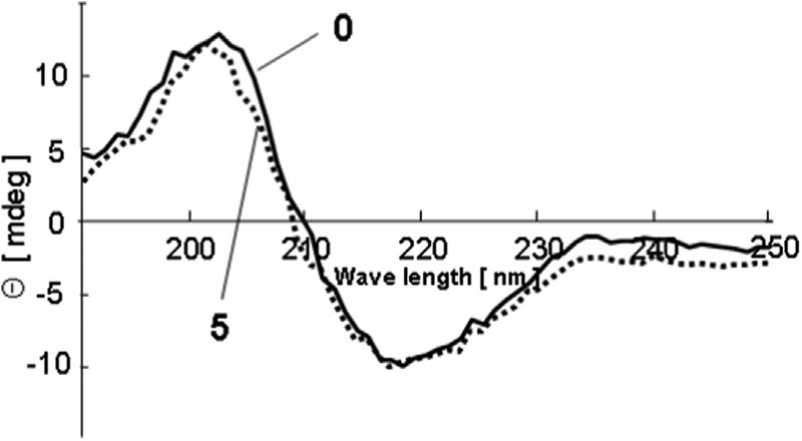
CD spectra of the mKate2 aqueous solution with no additives after 0 (solid line) and 5 (dotted line) cycles of drying-rehydration.

As shown in Fig. 2, the CD spectrum of the mKate2 aqueous solution without any additive was almost unchanged after five cycles of drying-rehydration. This indicates that mKate2 suffered from little or no secondary structural change on desiccation even without the aid of any protectants.

**Fig. 3:**
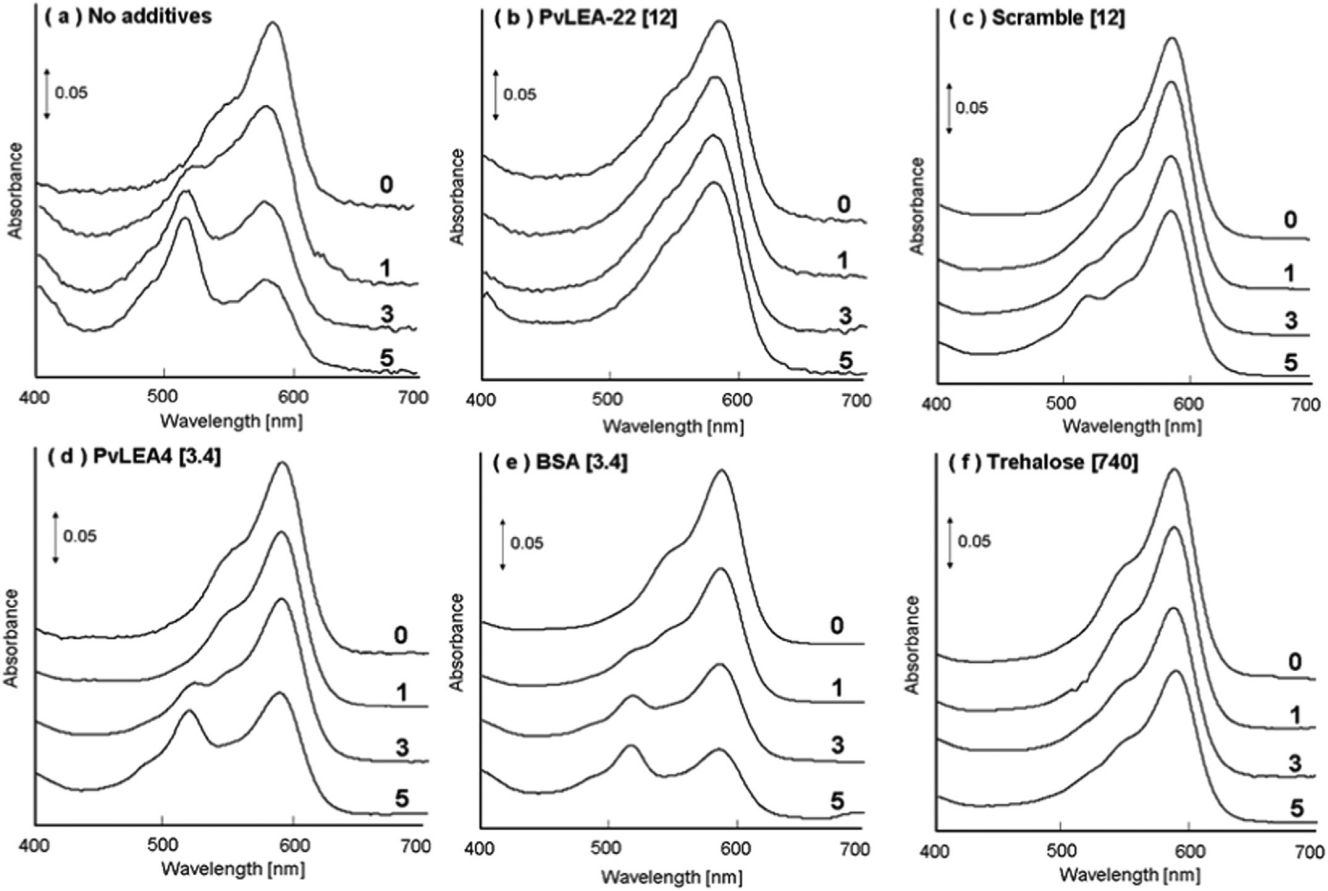
Absorption spectra of mKate2 aqueous solution after 0, 1, 3, and 5 cycles of drying-rehydration. Number in brackets indicates the molar ratio of each additive relative to mKate2.

Fig. 3 shows the results for the absorption spectral measurements. The mKate2 aqueous solutions before drying showed a main absorption peak at 588 nm (Fig. 3a). However, this peak was slightly shifted to the shorter wavelength side with repeated drying and rehydration, and concomitantly a new peak developed around 515 nm. This suggests that the structure of the mKate2 fluorophore is modified by the desiccation stress imposed. When PvLEA-22 was added to the mKate2 solution, on the other hand, the absorption spectra were almost unchanged throughout the five drying-rehydration cycles (Fig. 3b). Very similar results were obtained in the presence of the scrambled peptide, although a shoulder appeared at around 515 nm after the fifth cycle of drying-rehydration (Fig. 3c). Thus, the scrambled peptide may be somewhat less effective than PvLEA-22 as a protectant for mKate2. Unexpectedly, as far as we were able to judge from the absorption spectral changes, a native G3LEA protein, PvLEA4, was less effective than these model peptides: the 515 nm peak was clearly present after the fifth cycle of drying-rehydration (Fig. 3d). This peak was also observed in the BSA-containing solutions: in this case, it appeared already after the third cycle (Fig. 3e). When trehalose was added at a molar ratio of 74 relative to mKate2, the resulting spectral changes were similar to those without additives (Fig. 3a; data not shown). However, excellent protection was observed at the higher molar ratio of 740 (Fig. 3f): the 515 nm peak did not develop throughout the five drying-rehydration cycles, and thus the resulting spectra were very similar to those observed for the PvLEA-22-containing solution (Fig. 3b).

**Fig. 4:**
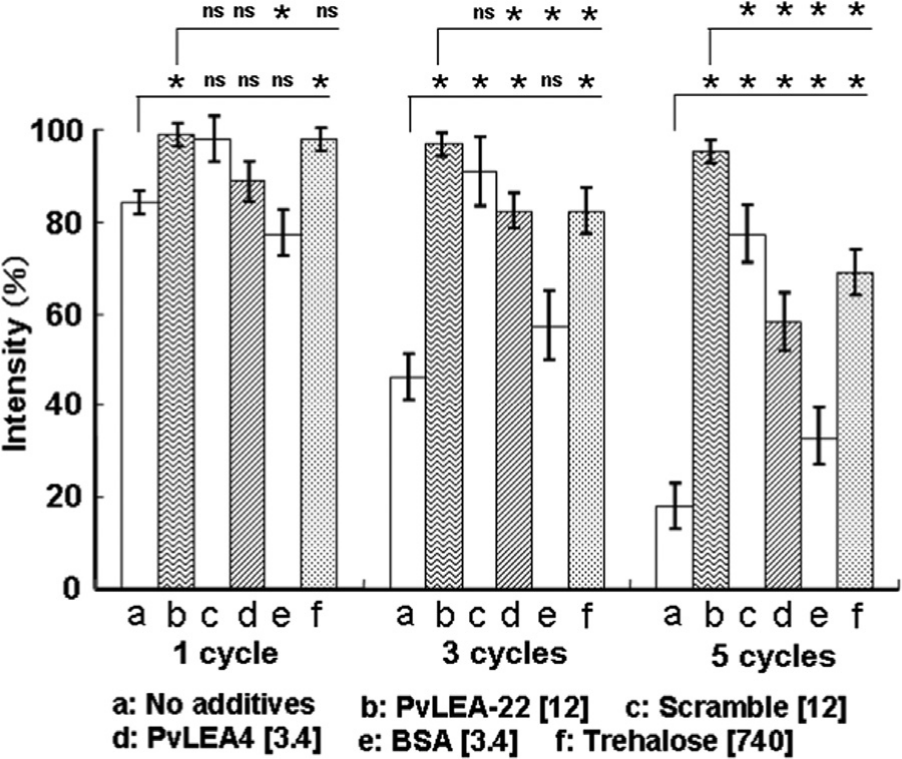
Relative fluorescence intensity of mKate2 aqueous solution after 1, 3, and 5 cycles of drying-rehydration. The intensity before drying (0 cycle) was defined as 100%. Each value represents mean ± SD (n = 3). *, P < 0.05; ns, not significant.

Fig. 4 shows the changes in fluorescence intensity of mKate2 with or without protectants. In the absence of any protectant, the fluorescence intensity decreased as the number of drying-rehydration cycles increased (bars labeled ‘a’). This is consistent with the decrease in the peak intensity at 588 nm in the absorption spectrum (Fig. 3a). Surprisingly, in the presence of PvLEA-22, the fluorescence intensity was maintained almost perfectly throughout the five drying-rehydration cycles (bars labeled ‘b’), being consistent with the absorption result (Fig. 3b). The scrambled peptide, PvLEA4 and a large molar ratio of trehalose exhibited weaker protective effects (bars labeled ‘c’, ‘d’ and ‘f’) compared with PvLEA-22. For BSA, no apparent protective effect was observed (bars labeled ‘e’). Therefore, among the protectants studied, PvLEA-22 was the most effective in the protecting the fluorescent center of mKate2 from desiccation-induced damage.

RFPs have the following structural features: 1) they have a cylindrical geometry, referred to as a β-can [35], which is made of eleven strands of β-sheet structure [33], [35], [36]; 2) one end of the β-can is capped by three short (5–8 residues) α-helical segments, and the other end is capped by one short α-helical segment, which is, however, very distorted [33], [35], [36]; and 3) the fluorophore is centrally located in the β-can, as a part of an α-helix which runs along the axis of the β-can [33], [35], [36]. The tightly constructed β-can structure is thought to be responsible for resistance to unfolding caused by heat and denaturants [35], [36]. Nevertheless, the RFP fluorophore apparently undergoes significant structural modifications during desiccation as is evident from the changes in absorption and fluorescence spectra shown in Figs. 3 and 4.

In its native configuration, the fluorophore is protected from collisions with fluorescence quenchers such as oxygen and/or solvent molecules [35], [36]. Accordingly, direct access to the fluorophore should not occur even for a short peptide such as PvLEA-22 and for a disaccharide such as trehalose. It is thus likely that these molecules may attenuate the physical stress imposed on the target protein by covering its outside surface, probably providing indirect protection of the fluorophore. The function of trehalose as a desiccation protectant is partially attributed to its ability to form sugar glasses and to replace the bound water strongly associated with the surface of proteins and with the headgroups of phospholipid bilayers [37], [38], [39]. Our previous studies on PvLEA-22 demonstrated that, similarly to trehalose, this peptide easily vitrifies in the dry state [26], [27] and directly interact with the head groups of a phospholipid bilayer in place of bound water [29]. Taken together, this would suggest that the vitrification and the water replacement mechanisms are probably also responsible for the preservation of the RFP fluorophore studied here.

The scrambled peptide was somewhat less effective than PvLEA-22. Our previous work has shown that PvLEA-22 is a kind of intrinsically disordered peptide: it is disordered in solution but forms α-helix in the dry state [26], [27]. In contrast, while the scrambled peptide is also disordered in solution, it does not fold on drying. This may explain the difference in protective ability of the two peptides. As described in the Introduction, Chakrabortee et al. demonstrated only limited protection of another RFP, mCherry, by a native G3LEA protein in the dry state [32]. To explain this result, they cited the entropy transfer model proposed by Tompa and Csermely [40] whereby intrinsically disordered LEA proteins might gain secondary structure on the surface of target proteins, potentially allowing a degree of unfolding followed by correct refolding of the target protein [32]. Such an entropy transfer model could also explain the protective effect of PvLEA-22. However, this model should be ruled out for a disaccharide like trehalose, which may at least partly explain why this sugar is less active than PvLEA-22.

According to the present study, a native LEA protein, PvLEA4, is less effective than the LEA model peptide, PvLEA-22. This result may be interpreted as follows. The pI values for PvLEA4 [12] and PvLEA-22 [28] are 5.4 and 7.2, respectively. The pI value of mKate2 is estimated to be approximately 5–6 from the pH titration curve given in Ref. [33]. Thus, PvLEA4 and mKate2 should both be negatively charged under physiological conditions, which implies there are repulsive interactions between PvLEA4 and mKate2 at neutral pH and thereby the close shielding of RFP by PvLEA4 might be electrostatically difficult. For a complete understanding of the functional difference between the model peptide and the native protein, it may be necessary to elucidate the structure and function not only of the 11-mer motif regions, but also of the residual non-repeating regions of native G3LEA proteins. This is now under investigation in our laboratory.

It is known that BSA has an anti-aggregation effect on aggregation-prone proteins: for example, in our previous study, BSA reduced aggregation in α-casein subjected to desiccation and rehydration [12]. In the present study, however, no apparent protective activity was observed for BSA. To maintain the precise three-dimensional structure of a target protein, close shielding of its surface would be required, which may be different to the requirements for inhibition of aggregation. In this regard, a large globular protein with fixed conformation, such as BSA, may be disadvantageous compared with IDPs or IDP-like peptides, which could be more flexible in their interactions with the target structure.

In summary, the LEA model peptide, PvLEA-22, is promising as a protective reagent for proteins that are prone to undergo three-dimensional structural change in the dry state.
